# To Use or Not to Use: No Consensus on Whether and How to Apply Genetic Information in the Justice System

**DOI:** 10.3390/bs9120149

**Published:** 2019-12-10

**Authors:** Fatos Selita, Robert Chapman, Yulia Kovas

**Affiliations:** 1 Department of Psychology, Goldsmiths, University of London, London SE14 6NW, UK; r.chapman@gold.ac.uk (R.C.); y.kovas@gold.ac.uk (Y.K.); 2 International Centre for Research in Human Development, Tomsk State University, Tomsk 634050, Russia

**Keywords:** genetic information, justice system, sentencing, free will

## Abstract

Little is known about the public’s attitudes towards applying genetic information in the justice system. This study aimed to extend previous research to explore this among the general public and those with training in law. Data were collected from over 10,000 participants, including 486 lawyers and law students. We analysed eight available relevant items from the International Genetic Literacy and Attitudes Survey (iGLAS). The majority of participants viewed genetic information as relevant to justice. For example, 65% believed that we should make provisions (legal and policy) to buffer the effects of genetic disadvantage on individuals, and almost 60% believed that genetic information should be taken into account in sentencing. At the same time, many participants (70%) disagreed that genetic influences on behaviour negate free will. The results of the correlational analyses suggest that people who consider genetic information relevant in one context tend to consider it relevant across all aspects of the justice system, including in sentencing, crime prevention and access to justice. Overall, the results suggest that views on the use of genetics by justice systems are complex and widely varied. Further research is needed to understand these complex views.

## 1. Introduction

New knowledge on the aetiology of human behaviour brings new challenges to ensuring justice [1,2]. Today we know that both genes and environments play a significant role in all human traits and that our actions are a product of complex gene–environment interplay [3,4,5]. Societies now need to develop policies and guidelines on how aetiological information should be used in the justice system. A starting point for this is evaluating people’s views on the aetiology of behaviour and on whether and how aetiological information should be used in court. The limited research that is available suggests that people hold diverging views on the implications of genetics to the concepts of free will, fairness and sentencing [6,7,8,9,10]. 

For example, one study of 250 U.S. adults presented participants with a vignette describing an apparently impulsive homicide, accompanied by one of four explanations of the defendant’s impulsivity. Participants assigned longer sentences for genetic risk and for the combination of genetic risk and abuse experienced in childhood than abuse or impulsivity alone [11]. Presumably this is because people view these factors as strong determinants, leading to risk of reoffending. Similar results were found in another recent study that included 218 participants [12]. Several studies also showed that environmental explanations (e.g., childhood abuse) led to overall longer sentence [11,13]. Somewhat paradoxically, this effect was mediated by the belief of greater control associated with environmental explanations.

Another study with 181 US state trial judges explored the use of aetiological explanations in sentencing [14]. The results showed that a mental disorder (psychopathy) was viewed by most judges as an aggravating factor in sentencing. However, when psychopathy was associated with a genetic explanation (biomechanism), the average sentence was reduced (from 13.93 years to 12.83 years). The study also found wide variation across the judges in the sentence length given in the same case, ranging from one to 41 years. Other studies have explored views of legal professionals and other people, and suggested the existence of multiple biases that underlie the observed variation in decisions [15,16]. 

Overall, the limited research available suggests that people’s views on aetiology of behaviour may underlie their decisions in regard to responsibility and sentencing. Moreover, sentencing decisions depend not only on individual factors, but on their combinations. For example, genetic evidence may be viewed as irrelevant to sentence; as having a mitigating effect; or as having an aggravating effect (for example, when genetic risk is associated with greater risk of reoffending). Most previous research relies on a few items or single vignettes, and therefore may leave unexplored many possible reasons for the wide variability in views and decisions. Moreover, it is important to explore people’s views on whether genetic evidence should be used at all in policing and the justice system.

### The Current Study

This study seeks to extend the existing research to explore people’s views on the use of genetic information in the justice system. We have utilised available data from a large unselected sample of adults, as well as a sample of lawyers and law students. We explored people’s views on the following five issues:

1. Do genetic influences on behaviour affect ‘free will’?
2. Is there ‘genetic disadvantage’ in the same way as there is ‘environmental (circumstantial) disadvantage’?
3. Should genetic disadvantage be taken into account in criminal sentencing, and if yes, how?
4. Should societies make provisions to buffer the effects of genetic disadvantage?
5. Should the state use genetic information for crime prevention?

We expected wide variability in people’s views on some issues, but more agreement on others. Specifically, we expected that most people would agree that there is genetic disadvantage and that this disadvantage should be accommodated by the legal system. We also expected that most people would disagree with the state using genetic information for crime prevention, as people would not want to endorse the state having access to personal data without consent.

## 2. Materials and Methods

### 2.1. Sample

Participants represented an opportunistic sample. They were reached through social media, Reddit Ask Me Anything (AMA) and by email (see [17] for further description of the sample). Data were collected online, therefore all participants were computer literate and had access to the internet; 88% of all respondents indicated that they had completed or were working towards university degree-level qualifications. The number of participants varied across different analyses, as not all participants answered all questions.

#### 2.1.1. Full Sample

A total of 13,356 participants initiated the survey. After removing outliers (i.e., ‘impossible age and other impossible values’; discontinued responses), the sample included 10,090 participants. The mean age was 30.16 (standard deviation (SD) = 11.21, range 18–80). Of these, 3643 (36.1%) were male; 6105 (60.5%) were female; 63 (0.6%) were non-binary; 69 (0.7%) preferred not to say; and 210 (2.1%) participants did not provide gender information.

#### 2.1.2. Law Sub-Sample

Except for the first three versions of the International Genetic Literacy and Attitudes Survey (iGLAS), additional items were included specifically for legal professionals and law students (collections from 17/08/2017 onwards). In total, 486 participants completed these additional law items. 217 were law students (age mean (M) = 22.40; SD = 4.13; range 18–54), 224 were law professionals (age M = 44.4; SD = 9.39; range 18–66) and 45 participants reported that they were both working in a legal profession and studying law (age M = 22.98; SD = 2.82; range 18–31). For the purposes of this study, lawyers and law students were grouped together. Gender distribution was very similar to the whole sample: 169 (36.1%) were male; 309 (62.1%) were female; 1 (0.4%) was non-binary; and 2 (1.5%) preferred not to say.

### 2.2. Procedure

The study was approved by the Goldsmiths Department of Psychology Ethics Committee, UK; and the Ethics Committee for Interdisciplinary Research of Tomsk State University, Russia. The online International Genetic Literacy and Attitudes Survey (iGLAS) was used to collect data (see [17,18]). iGLAS is a dynamic instrument, currently in its 10^th^ version, which consists of five parts: genetic knowledge, attitudes and opinions about genetics, demographic information, vignettes and neuromyths. iGLAS can be completed in seven languages, with some languages becoming available only at later collection waves. In this study, the majority of participants completed iGLAS in English (35.9%) and Russian (56.9%). Other languages were Italian (2.4%), Romanian (1.4%) and Spanish (3.4%). Participants completed the iGLAS online from their own devices/computers in their own time. Informed consent was implemented at the beginning of the survey. The completion of the survey took approximately 15–20 min. Data were collected between 31/10/2017 and 30/03/2019.

Questions are formatted in several ways to help reduce the effects of common method variance [19]. The latest version of the study can be found at http://tagc.world/iglas/. We previously reported analyses that focused on the genetic knowledge (GK) section, as well as six items from the attitudes section of iGLAS [17]. The present study analyses the results from eight previously unreported items, as described in the following section. All analyses were conducted using SPSS Version 23. 

### 2.3. Measures

The analyses evaluated responses on eight opinion items. Seven items were measured on a 7-point Likert scale, from strongly disagree to strongly agree. The exact items and participant numbers for Items 1–7 are presented in Table 1. The eighth item was a vignette for which participants selected one of the four response options.

The number of participants differed across items, because two items were presented to all participants; two items were only included in the later versions of the iGLAS; and four items were only presented to participants who identified either as lawyers and/or law students (using adaptive branching algorithm). The largest number of participants’ responses were available for the vignette, as this item was also included in earlier versions of iGLAS. Halfway through the data collection, one of the response options was replaced with another, which provided an interesting comparison for the present study.

## 3. Results

The data were evaluated using descriptive and correlational analyses.

### Descriptive Statistics

Table 1 presents descriptive statistics for the seven items (1–7) that had the same response options, as well as the numbers of participants who completed each item. The means and SDs are overall similar for Items 2–7, suggesting greater agreement with these statements; Item 1 generated the smallest mean, suggesting greater disagreement with the statement.

Table 2 presents frequencies of responses for each item on the 7-point Likert scale. The whole scale of the possible responses was used by the participants, showing wide variability in views. However, most participants agreed to some extent that: There is genetic disadvantage (Item 2) and that we should make provisions (legal and policy) to buffer the effects of genetic disadvantage on individuals (Item 3); genetic information should be taken into account in deciding the form (Item 5) and length (Item 6) of sentence; and that the legal system should accommodate differences among people (Item 7). Most participants disagreed that genetic influences on behaviour negate free will (Item 1); and endorsed the use of genetic information by the state for crime prevention (Item 4). 

Table 3 presents the correlation matrix among the seven items. Of the seven items, five correlated moderately positively with each other (Items 3–7).

Table 4 and Table 5 present the results for the vignette (Item 8). Table 4 presents frequencies of each of the four response options for the whole sample. 

We further analysed the data for this vignette splitting the sample into two groups: early iGLAS, *N* = 5566 and late iGLAS, *N* = 4216. Both groups had the same three response options, with the 4th response option being different. Table 5 presents frequencies for the two groups. Very few participants chose to ‘reduce’ (4.9% and 5.7% for samples 1 and 2) or ‘increase’ (0.6% and 2.5%) the sentence based on this genetic risk. For the group completing the earlier version of iGLAS, 52.4% opted for genetic information being considered in sentencing, but making no difference to the sentence; with the remaining 42% opting for it not to be taken into consideration. In the group presented the later iGLAS version, 62.1% opted for genetic information being considered to determine the type of sentence, with only 28% opting for ‘not be taken into consideration’.

## 4. Discussion

The results of the study confirmed our expectation of a wide variability in people’s views; the whole scale of responses was used by the participants for each of the eight items. The responses to the Item 1 (‘free will item’) may mean that most participants do not think that genes place significant limitations on free will. However, responses to Items 2, 3, 5 and 6 show that many of the same participants also think that both genes and environment have significant impact on human behaviour. In fact, over half of the participants agreed that *there is genetic disadvantage, in the same way as there is environmental disadvantage,* with only about a quarter of participants disagreeing (Item 2). 

Nevertheless, most participants seem to be reluctant to take such disadvantage into account in criminal sentencing. The pattern of responses to the vignette (Item 8)—asking participants to decide whether *having a genetic variant associated with aggression should be considered in a criminal case involving violent crime*—shows that most participants think that genetic risk should not affect the length of sentence. Analyses of the data from the two differing responses in the earlier and later versions of iGLAS showed an interesting pattern. Over half of the participants opted for the genetic risk to be considered by the court but make no difference to the sentence when this option was available, with most of the remaining participants (42%) opting for *‘Not be taken into consideration’.* Even more participants (over 60%) opted for genetic information to be considered to determine the type of sentence, when this option was available, with only 28% opting for *‘Not be taken into consideration’*.

These results suggest that many people believe that it is fair to consider genetic risk in criminal cases, presumably to acknowledge genetic disadvantage as unfair and to empathise with the disadvantaged. At the same time, the participants’ willingness to use genetic risk to affect sentence may stem from considerations of public safety. This is consistent with previous research that found harsher sentencing decisions when genetic aetiology was linked to reoffending or other additional risks [12,13]. In the present study, most people were willing to take genetic risk into account to determine the type of punishment when given such an option. This may be because people view this option as fairer (for example, preferring educational or medical interventions to prison sentence). Alternatively, as discussed above, people may think that genetic risk or a combination of different risks reduce control and therefore must result in prison sentence. Further research is needed to investigate these and other possible explanations for the pattern of results whereby genetic information is viewed as more relevant to type than to length of punishment.

Results from Items 5 and 6 are also consistent with most people’s willingness to consider genetic make-up to improve justice. Over half of the participants agreed that human behaviours are a product of multiple gene–environment processes, often beyond an individual’s control, and that this information should be taken into account in deciding the form and length of sentencing. Consistent with previous studies [13,15], it appears that when aetiological information is presented as more complex/multifactorial (rather than one genetic variant as in the vignette), more participants agree that aetiology should affect both form and length of sentence. However, additional items are required to investigate whether participants endorse the use of genetic information for increasing or decreasing the sentence. Previous research, described in the introduction (e.g., [11,15]), suggested wide variability in people’s views on whether genetic information should be viewed as mitigating or aggravating in different circumstances. Furthermore, as these items were only available for legally trained people in our study, it is not clear whether lawyers are more willing to take genetic information into account in sentencing than non-lawyers. We are currently conducting research to explore this further. 

As predicted, responses to Item 3 showed that the majority (64.8%) agreed that *we should make provisions (legal and policy) to buffer the effects of genetic disadvantage on individuals (e.g. tailored education)*. Presumably, participants mean that it is possible to prevent negative outcomes, such as crime. Further research is needed to clarify what effective provisions for buffering genetic disadvantage people envisage, when they endorse this item, beyond the provided example of tailored education. Most (71.3%) lawyers and law students also agreed (vs 16% who disagreed) that the legal system should accommodate differences among people in terms of ability, personality and level of education, including in terms of procedure and resources (Item 7). This is consistent with previous studies that, for example, endorsed greater genetic education among legal professionals and importance of expert support in cases involving genetics [1,20].

Contrary to our expectation, a large proportion of lawyers and law students (56.2%) agreed that the state *should use genetic information (e.g., propensity for violence) for prevention of crime*. Further research is needed to clarify what preventative measures participants would be prepared to endorse. For example, the state may use intrusive and coercive measures (e.g., surveillance on genetic basis; segregation); or measures that benefit individuals (e.g. such as pre-school socio-emotional educational programmes). 

Correlational analyses explored whether people’s views regarding using genetic information in the legal system are consistent across different contexts. The results suggest that people who agree that *‘We should make provisions (legal and policy) to buffer the effects of genetic disadvantage on individuals’* (Item 3), also tend to think that *genetic information should be used in determining the form* (Item 5) *and length* (Item 6) *of sentencing,* that *court procedures should be more people friendly* (Item 7)*,* and *that the state should use genetic information to prevent crime* (Item 4). The item *‘Genetic influences on our behaviour mean that there is no free will’* (Item 1) showed weak/negligible correlations with other items. This is likely because many people who did not agree with this statement nevertheless think that genetic effects are relevant to behaviour. There also may be some overlap between ‘somewhat agree’ and ‘somewhat disagree’ responses. Another item that was only weakly (and negatively) correlated to other items is: *‘In the same way as there is socio-economic disadvantage, there is genetic disadvantage’* (Item 2). This may be because of some ambiguity of the statement, as many people may agree that there are both types of disadvantage, but disagree that they are of the same weight in effect. Interestingly, this item showed the strongest negative correlations (−0.29 and −0.25, respectively) with the *State using genetic information* (Item 4) and *genetic information affecting the length of punishment* (Item 6). This might mean that some of the participants who believe that there is genetic disadvantage worry that using genetic information can exacerbate injustice; for example, through state surveillance on a genetic basis or longer prison sentence.

## 5. Conclusions

Overall, the results suggest that people’s views on the use of genetic information in the justice system are complex and varied. Many people believe that genetic information is relevant and should be taken into account in sentencing and other legal system provisions. People who endorse using genetic information in one justice context tend to also endorse such use in other aspects of the justice system. People who think that genetic disadvantage should be buffered by special provisions tend to think that the state should use genetic information to prevent crime. Further research is needed to investigate what state measures people would be prepared to endorse. More research is also needed to understand views on what factors and how strongly should affect the sentence, and how potential interactions between factors should be accounted for. 

A limitation of the current study is that some items were only presented to law professionals and law students. Future studies are needed to explore whether lawyers and non-lawyers differ in their considerations of genetic information in the justice system. The majority of participants in the current study indicated that they completed or were working towards university degree-level qualifications. Another feature of the sample is that most participants were Russian speakers (57%) or English speakers (36%). Further research is needed to explore potential group differences in views on the use of genetic information in the justice system, such as differences in education, culture, age and birth cohorts. 

## Figures and Tables

**Table 1:** Means, standard deviations and ranges for response to Likert scaled items.

No.	Item	N	Mean (Standard Deviation)	Range
1	Genetic influences on our behaviour mean that there is no free will.	4566	2.83 (1.66)	1–7
2	In the same way as there is socio-economic disadvantage, there is genetic disadvantage.	845	4.45 (1.52)	1–7
3	We should make provisions (legal and policy) to buffer the effects of genetic disadvantage on individuals (e.g., tailored education).	848	4.95 (1.46)	1–7
	Administered only to Law professionals and Law students			
4	If we find that people with certain genetic mutations have a propensity for violence, the state should use this information for prevention of crime.	473	4.38 (1.83)	1–7
5	According to the latest genetic findings, human behaviours are a product of multiple gene–environment processes, often beyond an individual’s control: This information should be taken into account in deciding the form of sentencing (e.g., compulsory therapy or education, community service, prison sentence).	420	4.47 (1.63)	1–7
6	According to the latest genetic findings, human behaviours are a product of multiple gene–environment processes, often beyond an individual’s control: This information should be taken into account in deciding the length of punishment.	418	4.06 (1.68)	1–7
7	Findings show that within any population there is a very large variability among people, including in terms of ability, personality and level of education. To provide justice for all, the legal system should accommodate this variability, including in terms of procedure and resources. For example, providing accessible jargon free information and making court proceedings people friendly.	426	5.06 (1.53)	1–7

**Table 2:** Responses to Likert scaled items: N and % of participants choosing each response.

No.	Item (Abbreviated Descriptions)	Strongly Disagree—Strongly Agree						
1	‘Genetics and free will’	1007 22%	1548 34%	632 14%	545 12%	361 7%	353 8%	120 3%
2	‘Genetic disadvantage’	28 3%	80 10%	109 13%	190 23%	204 24%	174 21%	60 7%
3	‘Buffering against genetic disadvantage’	17 2%	49 6%	60 7%	173 20%	195 23%	243 29%	111 13%
	Administered only to Law professionals and Law students							
4	‘Use of genetic information by the State’	45 10%	56 12%	45 10%	61 13%	104 22%	116 25%	46 10%
5	‘Genetic information in sentencing: form’	25 6%	47 11%	39 9%	58 14%	114 27%	118 28%	19 5%
6	‘Genetic information in sentencing: length’	40 10%	60 14%	47 11%	57 14%	123 29%	83 20%	8 2%
7	‘Legal system accommodating variability’	13 3%	27 6%	28 7%	54 13%	90 21%	161 38%	53 12%

Darker shading of the cells corresponds to a greater proportion of respondents selecting that specific option on the Likert scale. The central column indicates a response of neither agree nor disagree.

**Table 3:** Pearson correlations (*r*).

	Item (Abbreviated Description)		1	2	3	4	5	6	7
**1**	‘Genetics and free will’	*r*	1	0.09 **	0.03	0.14 **	0.08	0.13 **	−0.03
		N	4556	843	845	472	420	418	425
2	‘Genetic disadvantage’		*r*	1	−0.02	−0.29 **	−0.12*	−0.25 **	−0.06
			N	845	844	472	420	418	425
3	‘Buffering against genetic disadvantage’			*r*	1	0.48 **	0.46 **	0.43 **	0.38 **
				N	848	472	419	417	425
4	‘Use of genetic information by the State’				*r*	1	0.49 **	0.52 **	0.29 **
					N	473	419	417	425
5	‘Genetic information in sentencing: form’					*r*	1	0.58 **	0.39 **
						N	420	416	420
6	‘Genetic information in sentencing: length’						*r*	1	0.32 **
							N	418	418
7	‘Legal system accommodating variability’							*r*	1
								N	426

Strength of correlations indicated using a heat map: darker shades correspond to stronger correlations. Some items have been paraphrased for ease of reference. * Correlation significant at the *p* = 0.05 level; ** Correlation significant at the *p* = 0.01 level.

**Table 4:** Responses to the vignette (Item 8): Sarah has a particular genetic variant that has been associated with aggression. She is in court being tried for a violent crime. Should knowing about this genetic variation: (select one of the 4 options).

	Frequency	Percent
Reduce her sentence	526	5.2
Not be taken into consideration	3554	35.2
Increase her sentence	146	1.4
Be considered but make no difference to her sentence ^a^	2974	29.5
Be considered to determine the type of sentence (e.g., mandatory labour, psychological therapy) ^b^	2782	27.6
Total	9982	98.9
Missing	108	1.1
Total	10,090	100.0

^a^ This response option was presented in the International Genetic Literacy and Attitudes Survey (iGLAS) versions 1.1, 1.2, 1.3, 2.1 and 2.2.; ^b^ This response option was presented in iGLAS versions 2.2, 2.3, 2.4, 2.5 and 2.6.

**Table 5:** Vignette responses for different versions of iGLAS with differing response options.

Response	Earlier iGLAS	Later iGLAS
Reduce her sentence	4.9% (275)	5.7% (245)
Not be taken into consideration	42% (2338)	28% (1200)
Increase	0.6% (36)	2.5% (106)
Be considered but make no difference to the sentence ^a^	52.4% (2917)	-
Be considered to determine the type of sentence (e.g., mandatory labour, psychological therapy) ^b^	-	62.1% (2665)

^a^ and ^b^—response options presented in earlier and later iGLAS versions, respectively.
